# Predictors of cardiovascular events in multiple myeloma: a systematic review and exploratory meta-analysis

**DOI:** 10.3389/fcvm.2026.1925889

**Published:** 2026-10-20

**Authors:** Tiantian Lv, Minjie Liu, Weiwei Zhang, Jie Zhang, Yun Fang

**Affiliations:** 1 Union Hospital, Tongji Medical College, Huazhong University of Science and Technology, Wuhan, China; 2 School of Nursing, Tongji Medical College of Huazhong University of Science and Technology, Wuhan, China; 3 Yangtze University Health Science Center, Jingzhou, China

**Keywords:** cardiovascular events, exploratory meta-analysis, multiple myeloma, predictors, systematic review

## Abstract

**Background:**

Novel therapies have significantly improved survival in patients with multiple myeloma (MM), but cardiovascular (CV) events have become a major challenge, impairing treatment continuity and increasing mortality. This systematic review aimed to identify predictors of CV events in MM to support risk stratification and cardio-oncology management.

**Methods:**

This review followed PRISMA guidelines and was registered in PROSPERO (CRD420251084727). Five electronic databases were searched from inception to August 2025. Studies reporting multivariable predictors of CV events in MM were included. Methodological quality was assessed using QUIPS tool.

**Results:**

Eleven studies were included, comprising nine retrospective cohort studies, one prospective observational study, one case-control study. Overall, most studies were judged to be at moderate risk of bias. A total of 20 candidate predictors of CV events were identified. Among these, six were reported in at least two studies, but only hypertension had sufficiently comparable adjusted effect estimates for exploratory meta-analysis. Among treatment-related factors, carfilzomib-based therapy and longer duration of proteasome-inhibitor treatment were associated with a higher risk of CV event in individual studies. Limited evidence from a single prospective study suggests that dynamic natriuretic peptide assessment may have prognostic value, with elevation of natriuretic peptides during the first treatment cycle showing a stronger predictive value (HR 9.5) compared to baseline elevation alone (HR 4.1).

**Conclusions:**

CV risk in MM appears to be multifactorial, involving both pre-existing vulnerability and treatment-related toxicity. Although several candidate predictors have been identified, the current evidence remains limited by heterogeneity and methodological weaknesses. Larger prospective multicenter studies are required to validate these predictors and to define standardized CV risk assessment and surveillance strategies in MM.

**Systematic Review Registration:**

identifier CRD420251084727.

## 1 Background

Multiple myeloma (MM) is a malignant plasma cell disorder characterized by clonal proliferation of abnormal plasma cells in the bone marrow and secretion of monoclonal immunoglobulins or their fragments (M protein), leading to related organ or tissue damage (1). MM accounts for approximately 10% of all hematologic cancers, and the global incidence is rising (2). With advances in treatment and the introduction of novel agents, the 5-year survival rate has been significantly improved from 32% during 1995–1997 to 61% in the timeframe of 2014–2020 based on Surveillance, Epidemiology, and End Results (SEER) database statistics (3).

Given the incurable nature of MM (4), patients with MM inevitably undergo repeated exposure to various treatment regimens to derive benefits from therapeutic agents, while also bearing the corresponding complications, including CV events (5). CV events have emerged as an important challenge in MM, particularly among patients receiving proteasome inhibitor–based therapy (6, 7). A retrospective cohort study (8) indicated that compared with age and gender-matched non-cancer control groups, MM patients experience more frequent CV events.

Furthermore, newly diagnosed MM patients with comorbid CV diseases have increased all-cause mortality and MM-specific mortality (9), underscoring the clinical importance of CV management in this population. In recent years, increasing attention has been paid to factors associated with CV events in MM. Existing evidence suggests that CV risk in MM may arise from three broad domains: disease-related mechanisms, patient-related vulnerability, and treatment-related toxicity (5, 10, 11).

However, due to differences in study populations, sample sizes, treatment exposures, and outcome definitions, reported findings remain inconsistent. Therefore, this systematic review aimed to identify predictors of CV events in patients with MM.

## 2 Methods

This systematic review and meta-analysis was conducted in accordance with PRISMA 2020 guidelines (12, 13) and prospectively registered in PROSPERO (CRD420251084727).

### 2.1 Information sources and search strategy

A systematic literature search was performed in August 2025 in the following electronic databases: PubMed, Embase, Web of Science Core Collection, the Cochrane Library and Ovid MEDLINE. Searches were conducted from database inception to August 2025 using a combination of Medical Subject Headings (MeSH) terms and free-text keywords related to MM, CV events, and predictors. No language restrictions were applied to the database searches. However, because of feasibility constraints, eligibility at the full-text stage was restricted to articles available in English. Grey literature and conference abstracts were not included because multivariable-adjusted prognostic data were generally insufficient for full appraisal. The detailed search strategy for each database is provided in Supplementary Table S1.

### 2.2 Eligibility criteria

Eligibility criteria (Supplementary Table S2) were predefined according to the study population, predictors, outcomes, and study design. Studies were included if they enrolled adult patients (≥18 years) with MM diagnosed according to the 2014 IMWG criteria (1) or other contemporaneous diagnostic or registry-based definitions, investigated at least one clearly defined predictor of CV events, reported clearly defined CV outcomes, and provided quantitative effect estimates from multivariable analyses. Eligible study designs included prospective or retrospective cohort studies, case-control studies, and randomized controlled trials. Studies were excluded if they involved patients with concurrent cardiac amyloidosis, animal or *in vitro* models, lacked sufficient outcome data, reported only univariable analyses, or were non-primary publications such as reviews, case reports, editorials, conference abstracts, posters, or duplicate reports. Studies specifically enrolling patients with cardiac light-chain (AL) amyloidosis, or studies in which outcomes for MM could not be distinguished from those of patients with concomitant cardiac amyloidosis (CA) were excluded because cardiac involvement in AL amyloidosis represents a distinct and high-risk clinical entity with substantially worse prognosis (14), which could confound the assessment of cardiovascular outcomes in multiple myeloma.

### 2.3 Study selection

Two reviewers independently screened the titles and abstracts of all identified records according to the predefined eligibility criteria. Full-text articles deemed potentially relevant were subsequently assessed for inclusion. Any disagreements were resolved through discussion or consultation with a third reviewer. Studies were considered eligible if they enrolled patients with MM, examined demographic, clinical, treatment-related, and/or laboratory predictors of CV outcomes, and reported multivariable-adjusted effect estimates. Eligible studies were required to report clearly defined symptomatic or asymptomatic CV outcomes, which were grouped into: (1) major CV events; (2) treatment-emergent CV adverse events; (3) CV mortality; and (4) specific events such as hypertension or heart failure. Furthermore, CV outcomes were required to be ascertained based on clinical manifestations, imaging findings, laboratory evidence, or other standardized diagnostic criteria.

### 2.4 Risk of bias assessment

The methodological quality of the included studies was assessed using the Quality in Prognosis Studies (QUIPS) tool (15). The main concerns arose in the domains of study confounding, outcome measurement, and statistical analysis and reporting. In particular, adjustment for important confounders was inconsistent across studies, and variables such as baseline CV comorbidity, renal function, MM disease severity, and cumulative treatment exposure were not uniformly considered in multivariable models. In addition, CV outcomes were defined and ascertained differently across studies, which may have introduced variability in outcome measurement. Reporting of missing or incomplete data handling was also limited in several studies. Overall, these methodological limitations should be taken into account when interpreting the identified predictors and the pooled estimates. The certainty of evidence for the exploratory meta-analysis was assessed using an adapted GRADE framework for prognostic-factor evidence. Judgements considered risk of bias, inconsistency, indirectness, imprecision, and publication bias. Risk-of-bias judgements were informed by the QUIPS assessment. Because fewer than 10 studies were available for the quantitative synthesis, publication bias could not be reliably assessed.

### 2.5 Data extraction

The following information was extracted: (1) study characteristics, including first author, publication year, country or region, and study design; (2) participant characteristics, including age and sex distribution; (3) candidate prognostic factors, including demographic, clinical, treatment-related, and laboratory variables; and (4) CV outcomes and multivariable-adjusted effect estimates.

### 2.6 Data synthesis and statistical analysis

Given the substantial clinical and methodological heterogeneity across the included studies, including differences in study design, patient populations, CV outcome definitions, treatment exposures, follow-up duration, and reported effect measures, a single quantitative synthesis of all predictors was not considered appropriate. Therefore, the primary approach of this review was narrative synthesis. Adjusted effect estimates derived from multivariable models, including hazard ratios (HRs) and odds ratios (ORs), with corresponding 95% confidence intervals (CIs), were preferentially extracted. Predictors were categorized as patient-related, disease-related, treatment-related, or biomarker-related factors.

Meta-analysis was performed only for predictors reported in at least two independent studies with sufficiently comparable definitions, outcome ascertainment, and effect measures. Predictors that could not be pooled because of limited study numbers, inconsistent effect measures, heterogeneous variable definitions, or lack of extractable data were summarized narratively. For repeatedly reported predictors that were not pooled, adjusted effect estimates, 95% CIs, *P* values or statistical significance, direction of association, and consistency across studies were compared. For eligible predictors, pooled HRs and 95% CIs were estimated using a random-effects model. Heterogeneity was assessed using Cochran's *Q* test and the I^2^ statistic, with I^2^ > 50% or *P* < 0.10 indicating substantial heterogeneity (16). Because fewer than 10 studies were available for each meta-analysis, publication bias was not assessed. All analyses were conducted using Review Manager (RevMan), version 5.3, and a two-sided *P* value <0.05 was considered statistically significant. In the present review, hypertension was the only predictor that met the prespecified criteria for quantitative synthesis and was pooled using a random-effects model. Male sex was reported in more than one study but was not pooled because of differences in cohort definitions, cardiovascular outcomes, and analytical approaches. However, this pooled estimate was interpreted cautiously in view of the small number of eligible studies and differences in clinical context across the included reports.

## 3 Results

The study selection process is shown in Figure 1. The database searches identified 4,786 records in August 2025. After duplicate removal and title/abstract screening, 55 full-text articles were assessed for eligibility, of which 11 met the inclusion criteria and were included in the review (17–27).

**Figure 1:**
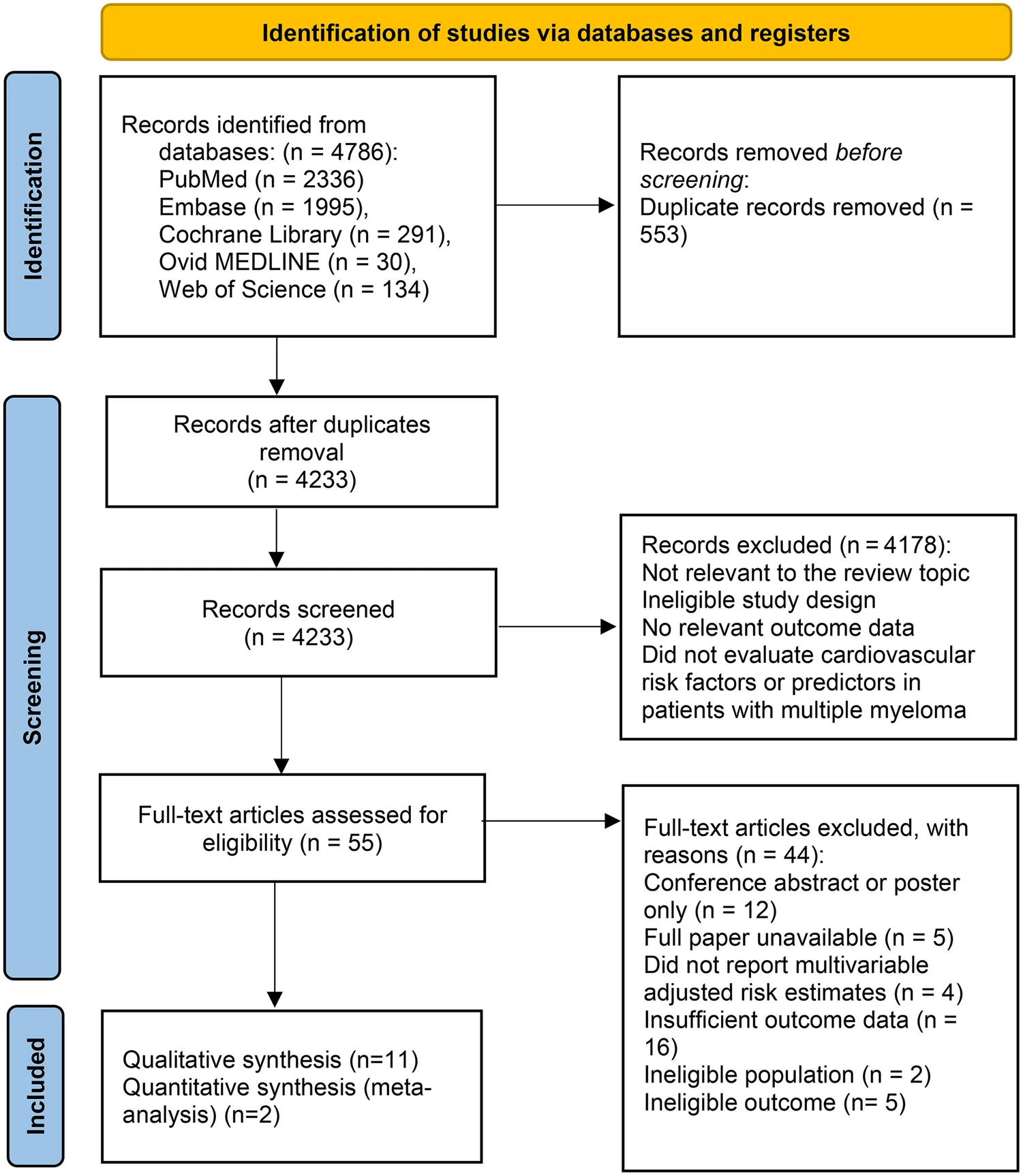
PRISMA 2020 flow diagram of study identification, screening, and inclusion.

### 3.1 Study characteristics

Characteristics of the included studies are summarized in Table 1. Most studies (17–24, 27) were retrospective cohort studies. One study (26) was a case-control study, one study (25) was a prospective observational study. These studies were conducted across four countries: the United States (19–23, 25–27) (*n* = 8), China (17) (*n* = 1), South Korea (18) (*n* = 1), and Germany (24) (*n* = 1). Five studies (20–23, 27) were population-based retrospective cohort studies derived from nationwide administrative or tumor registries, while remaining six studies (17–19, 24–26) were single-center hospital-based investigations.

**Table 1:** Main characteristics of the included studies.

First author, publication year	Country	Timeframe/setting	Study design	Sample size	Study population	Treatment regimen	Cardiovascular outcome	Follow-up	Evaluated predictors	Effect measure
Chari, 2016 (27)	United States	2005-2014; Truven MarketScan claims database	Retrospective cohort study	7,895 newly treated MM patients and 23,685 age- and sex-matched non-MM controls	Adults with newly diagnosed and newly treated MM; patients with MGUS and asymptomatic myeloma were excluded; median age 64 years, 55.7% male	Newly-initiated antimyeloma treatment; specific regimens not available in source data	Incident or worsening hypertension; malignant hypertension was additionally assessed	Median follow-up approximately 2 years	Age, baseline comorbidities, Charlson Comorbidity Index, baseline cardiovascular comorbidities	Univariate and multivariable Cox proportional-hazards models; HRs with 95% CIs
Chen, 2017 (26)	United States	2011-2014; Vanderbilt University Medical Center	Retrospective case-control comparison	96	Consecutive MM patients receiving proteasome inhibitor therapy; patients with AL amyloidosis were excluded; median age approximately 61 years; approximately 65% male	Bortezomib-based therapy *n*=44; carfilzomib-based therapy *n*=52; all carfilzomib-treated patients had prior bortezomib exposure	Composite cardiovascular adverse events: heart failure, hypertension, VTE, acute coronary syndrome, arrhythmia, pulmonary hypertension	Median time-to-event: 67.5 days; total follow-up NR	Prior cardiac events, duration of PI therapy, therapeutic agent, baseline cardiovascular comorbidities	Multivariable logistic regression; ORs with 95% CIs reported; cumulative incidence between bortezomib and carfilzomib compared using log-rank test
Backs, 2019 (24)	Germany	2004-2011; University Hospital of Würzburg	Retrospective cohort study	325	Patients with MM who underwent at least one autologous PBSCT; median age 61 years (IQR 55–67); 60.9% male	Autologous PBSCT; induction treatment included bortezomib-based, IMiD-based and non-novel-agent-based regimens, with specific regimens incompletely detailed	Primary outcome: all-cause mortality. Cardiovascular causes of death were described; echocardiographic cardiac function was assessed in a subgroup with available serial echocardiogram	Median follow-up 36 months (IQR 21–52)	Arterial hypertension, smoking history, age, sex, disease stage including Durie–Salmon stage B, treatment characteristics, baseline cardiovascular comorbidities and other clinical factors; echocardiographic parameters assessed in a subgroup	Univariate and multivariable Cox proportional-hazards models for mortality; HRs with 95% CIs reported
Cornell, 2019 (25)	United States	2015-2018; Vanderbilt University Medical Center & University of Pennsylvania Abramson Cancer Center	Prospective, observational, multicentre study	95	Patients with relapsed MM; median age 66 years; approximately 66% male	Carfilzomib-based therapy (*n*=65) or bortezomib-based therapy (*n*=30)	Composite cardiovascular adverse events, including heart failure, cardiac chest pain, hypertension, arrhythmia, acute coronary syndrome, pulmonary hypertension and VTE	Median follow-up among survivors: 25 months (range 3–41.8 months)	Treatment group, elevated baseline BNP/NT-proBNP, BNP/NT-proBNP increase 2–3 weeks after treatment initiation, time from diagnosis to treatment, and traditional CV risk-factor burden (≥2 vs. 0–1 factors)	Fine–Gray competing-risk subdistribution regression, including clustered-data analysis; SHRs with 95% CIs reported
Fakhri, 2020 (23)	United States	SEER–Medicare linked database; MM cases identified from 2000 to 2013, with claims available through 2014; carfilzomib exposure occurred in 2013–2014	Retrospective cohort study	635 carfilzomib-treated patients	Patients with MM treated with carfilzomib; median age 72 years (range 36–94); 55% male; 79% Caucasian	Carfilzomib monotherapy or carfilzomib with corticosteroids; patients receiving other concomitant anti-myeloma agents were excluded	Composite cardiac adverse events: hypertension, ischemic heart disease, or congestive heart failure	Cardiac events assessed from the first carfilzomib dose through 28 days after the last dose or initiation of subsequent anti-myeloma therapy; median carfilzomib treatment duration 58 days (range 1–716); median OS from carfilzomib initiation 13.1 months	Pre-existing chronic obstructive pulmonary disease, baseline comorbidities	Multivariable Cox proportional-hazards regression; adjusted HRs with 95% CIs reported
Bishnoi, 2021 (22)	United States	2001-2015; SEER-Medicare database	Retrospective cohort study	7,330 patients with MM, including 815 carfilzomib users	Medicare beneficiaries with MM, generally aged ≥66 years; 52% male	Carfilzomib-containing therapy; once-weekly or twice-weekly dosing schedules	Composite cardiovascular adverse events: heart failure, ischaemic heart disease, hypertension, oedema, syncope, dyspnoea, chest pain	Median time to CVAE onset among carfilzomib users, 3.1 months	Age > 75 years, pre-existing cardiovascular disease, pre-existing hypertension, obesity, twice-weekly carfilzomib schedule	Multivariable Cox proportional-hazards model; HRs with 95% CIs reported
Yin, 2022 (21)	United States	1975-2016; SEER database	Retrospective cohort study	88 328	Patients with MM identified in the SEER database; 53.25% male	Treatment regimens not available in source data	Cardiovascular disease mortality, including heart disease, hypertension without heart disease, cerebrovascular disease, atherosclerosis, and other arterial diseases	Median follow-up not reported; mortality analyzed by time since MM diagnosis	Male sex, Black race, advanced age at diagnosis, earlier diagnosis calendar era	SMRs for comparison with the general population; multivariable Cox regression with HRs for internal prognostic analysis
Chen, 2023 (20)	United States	2000-2015; SEER database	Retrospective cohort study	32 528	MM survivors with survival > 36 months; mean age 64.96 ± 11.00 years; 55.05 % male	Chemotherapy and radiotherapy were assessed; specific regimens not available	Cardiovascular disease mortality	NR	Age, sex, period of diagnosis, marital status, race, income, chemotherapy, and radiotherapy	Multivariable Cox proportional hazards and Fine-Gray competing-risk models; HRs and sHRs reported
Doran, 2023 (19)	United States	2011-2020; University of Maryland Medical Center	Retrospective single-centre cohort study	161	MM patients treated with carfilzomib; multi-racial cohort (53.4 % Black, 46.6 % White; median age 60 years)	Carfilzomib-based therapy	Composite cardiovascular adverse events: left-ventricular dysfunction, arrhythmia, myocardial infarction, pulmonary hypertension, dyspnoea with elevated NT-proBNP, new/worsening hypertension	NR	Hypertension, smoking history, race, sex, age, baseline echocardiographic findings, and cardiovascular risk burden	Multivariable logistic regression; adjusted ORs reported
Dai, 2024 (17)	China	2020-2023; Guangdong Provincial People’s Hospital	Retrospective observational study	47	MM patients meeting eligibility criteria for ASCT and with pre-/post-induction cardiac data; mean age 55.68 ± 11.38 years; 46.81% male	MM induction chemotherapy; exact PI-based combination regimens incompletely specified	Covert cardiovascular toxicity/cardiac injury related to induction chemotherapy, assessed by structural remodeling, electrical remodeling, and biomarker changes	NR; assessed at baseline, after induction chemotherapy, and before ASCT	*Δ*QT-interval (pre- vs. post-chemotherapy), light-chain subtype, induction-chemotherapy duration, blood urea nitrogen changes and other baseline clinical variables	Binary logistic regression; ORs reported
Jang, 2024 (18)	South Korea	2007-2019; Seoul National University Hospital	Retrospective cohort study	395	MM patients receiving bortezomib-containing therapy; mean age 67.6 years; 54.4 % male	Bortezomib-containing therapy	Composite cardiovascular adverse events: hypertension, arrhythmia, heart failure, myocardial infarction, pulmonary arterial hypertension, angina, VTE	At least 6 months to 2 years; median 730 days (IQR 658–730)	Low body-mass index, light-chain-type and IgD-type MM, pre-existing stroke, pre-existing hypertension	Multivariable Cox proportional hazards regression; adjusted HRs reported

NR, not reported; MM, multiple myeloma; MGUS, monoclonal gammopathy of undetermined significance; PI, proteasome inhibitor; IMiD, immunomodulatory drug; PBSCT, peripheral-blood stem-cell transplantation; ASCT, autologous stem-cell transplantation; CV, cardiovascular; CVAE(s), cardiovascular adverse event(s); HF, heart failure; VTE, venous thromboembolism; BNP, B-type natriuretic peptide; NT-proBNP, N-terminal pro-B-type natriuretic peptide; COPD, chronic obstructive pulmonary disease; HR, hazard ratio; aHR, adjusted hazard ratio; OR, odds ratio; SHRs, subdistribution hazard ratios; SMR, standardized mortality ratio; IQR, interquartile range.

Sample sizes varied substantially, ranging from 47 to 88,328 patients. The included studies collectively represented approximately 130,000 patients with MM, although some degree of overlap across registry-based populations could not be fully excluded. Several studies focused on specific clinical subgroups, including newly treated patients (27), patients receiving carfilzomib (19, 22, 23), and those undergoing autologous peripheral blood stem cell transplantation (24). The reported mean or median age ranged from 55.68 to 72 years, and the proportion of male participants ranged from 37% to 67%. The number of CV events ranged from 23 to 1,892, and the reported incidence of CV events ranged from 8.3% to 37.6%.

CV outcomes were heterogeneous. Six studies (18, 19, 22, 23, 25, 26) reported on cardiovascular adverse events (CVAEs) as the primary outcome, whereas three studies (20, 21, 24) focused on CV mortality and two studies (17, 27) reported on CV complication. The outcome assessments were either carried out with systematic surveillance (e.g., serial echocardiography, electrocardiography, cardiac biomarker testing) (*n* = 5) (17, 18, 24–26) or based on clinical event documentation and standardized coding (ICD codes for CV death, CTCAE 5.0 criteria for CVAEs) (*n* = 6) (19–23, 27).

Predictors were assessed at baseline in 4 studies (20, 21, 24, 27) and during treatment in 7 studies (17–19, 22, 23, 25, 26). In the included studies(*n* = 11) (17–27), demographic, clinical, and laboratory factors were investigated as potential risk factors or predictors for CV events. Predictor assessments covered three core categories: 1) demographic factors: age (20–22, 27), male sex (20, 21), race (20, 21), income (20); 2) MM-specific factors: disease subtype (18), stage (24), proteasome inhibitor treatment (25, 26) and 3) CV/comorbid factors: hypertension (18, 19, 22, 24), smoking history (19, 24), obesity (22), pre-existing cardiac disease (22, 25–27). Explicit methods for handling missing or incomplete data were reported in some studies (20, 21, 23), typically through exclusion of cases with unknown key variables, whereas several studies (17–19, 22, 24–27) did not clearly describe such methods. Substantial methodological and outcome heterogeneity was observed across the included studies.

### 3.2 Risk of bias assessment

QUIPS assessment showed that most studies were at moderate risk of bias, while registry-based studies and one prospective observational study were judged to be at relatively lower risk Table 2). The most frequent limitations were retrospective design, heterogeneous definitions of CV outcomes, and incomplete adjustment for treatment-related and CV confounders.

**Table 2:** Domain-specific risk of bias assessment using the QUIPS tool.

Study	Study participation	Study attrition	Prognostic factor measurement	Outcome measurement	Study confounding	Statistical analysis/reporting	Overall
Chari 2016 (27)	M	M	M	M	M	L	M
Chen 2017 (26)	M	M	M	M	H	M	M
Backs 2019 (24)	M	M	L	M	M	L	M
Cornell 2019 (25)	L	L	L	L	M	L	L
Fakhri 2020 (23)	L	L	M	M	M	L	M
Bishnoi 2021 (22)	L	L	M	M	M	L	M
Yin 2022 (21)	L	L	M	M	M	L	M
Chen 2023 (20)	L	L	M	M	M	L	M
Doran 2023 (19)	M	M	M	L	M	L	M
Dai 2024 (17)	H	M	M	M	H	H	H
Jang 2024 (18)	M	M	M	M	M	M	M

L, low risk of bias; M, moderate risk of bias; H, high risk of bias. Domain-level judgments were made according to the QUIPS framework across six domains: study participation, study attrition, prognostic factor measurement, outcome measurement, study confounding, and statistical analysis/reporting.

### 3.3 Reported predictors of cardiovascular events in patients with MM

A total of 20 demographic, clinical, therapeutic, and laboratory variables were identified as candidate predictors of CV events across the 11 included studies. Given the clinical and methodological heterogeneity in study design, CV outcome definitions, follow-up durations, and effect measures, meta-analysis was only conducted for factors that were consistently reported by at least two studies with comparable adjusted hazard ratios (HRs). Factors with inconsistent effect measures (e.g., HR vs. odds ratio [OR]) or insufficient comparability in definitions were synthesized narratively.

#### 3.3.1 Classification of cardiovascular outcomes

For predictor synthesis, cardiovascular outcomes were grouped according to the primary endpoint of each study: composite CVAEs, cardiovascular mortality, and cardiovascular complication/cardiotoxicity. Although these categories have been summarized in the study characteristics, they were used here to determine whether effect estimates were sufficiently comparable for quantitative pooling. The main source of heterogeneity was the definition of composite CVAEs. Across studies, these endpoints included different combinations of heart failure, hypertension, ischemic events, arrhythmia, pulmonary hypertension, venous thromboembolism (VTE), dyspnoea, oedema, syncope, and chest pain. Cardiovascular mortality studies also differed in cohort selection and analytical methods. Consequently, most predictors were summarized narratively. Hypertension was the only predictor with comparable adjusted hazard ratios from at least two studies evaluating composite CVAEs and was therefore retained for exploratory meta-analysis.

#### 3.3.2 Exploratory meta-analysis of hypertension for composite CVAEs

Hypertension was reported as a potential predictor of CV events in four studies (18, 19, 22, 24), three (18, 22, 24), studies reported hazard ratios (HRs), whereas one (19) reported an odds ratio (OR). One of the HR-based studies (24) modeled all-cause mortality rather than incident CVAEs and was therefore not eligible for pooling for this endpoint. To avoid inappropriate pooling of different effect measures, the meta-analysis included only two studies reporting HRs for incident composite CVAEs. The pooled estimate showed that hypertension was associated with an increased risk of CV events (**pooled HR** **=** **1.90, 95% CI 1.57–2.29; I^2^** **=** **0%, P for heterogeneity = 0.83) (**Figure 2**)**. Using an adapted GRADE approach for prognostic evidence, the certainty of this estimate was rated as low, mainly because of observational study design and indirectness in composite endpoint definitions (Supplementary Table S3). Doran et al. (19) reported an adjusted OR of 2.50 (95% CI: 1.10–5.90), which showed a consistent directional association with CV events and was thus included in the narrative synthesis. Because only two studies were pooled and their treatment settings and composite CVAE definitions were not identical, this result should be viewed as exploratory.

**Figure 2:**
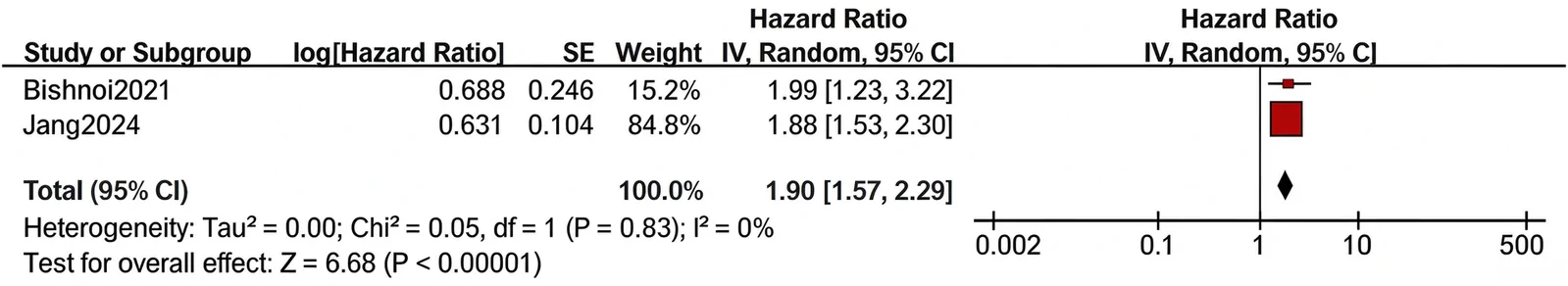
Exploratory meta-analysis of hypertension as a predictor of composite cardiovascular adverse events in patients with multiple myeloma.

#### 3.3.3 Demographic and lifestyle-related predictors

Other demographic and clinical predictors were narratively synthesized because of heterogeneity in outcome definitions, statistical models, and effect measures. Among demographic factors, male sex was associated with higher cardiovascular mortality in both SEER-based studies (20, 21), these estimates were not pooled owing to differences in cohort definition and analytic approach, but the direction of effect was consistent.

Advanced age was repeatedly reported as a risk factor for CV events across four studies (20–22, 27), however, quantitative pooling of effect estimates was not feasible due to inconsistent age stratification and reporting formats across studies. Smoking history was associated with adverse outcomes in two studies (19, 24), although the endpoints and effect measures differed. Doran et al. (19) reported an association with composite CVAEs (adjusted OR 2.80, 95% CI 1.30–6.40), whereas Backs et al. (24) reported an association in a mortality analysis (HR 2.13, 95% CI 1.12–4.05). Because these estimates referred to different outcomes and effect measures, they were not quantitatively compared. Two studies (18, 22) assessed body mass index (BMI) and reported that both underweight status (BMI <18.5 kg/m^2^) and obesity were independently associated with increased CV risk in MM patients.

#### 3.3.4 Clinical and comorbidity-related predictors

Both pre-existing and current CV disease were consistently associated with an increased risk of subsequent CV events in MM patients. Four studies (22, 25–27) investigated the association between a history of CV disease and CV event risk, and all reported a positive direction of effect. Other notable clinical predictors included chronic obstructive pulmonary disease (COPD) (23) (aHR 1.40, 95% CI: 1.03–1.90), peripheral arterial occlusive disease (PAOD) (24) (HR 10.4, 95% CI: 1.23–89.1), advanced Salmon-Durie stage B (24) (HR 2.28, 95% CI: 1.30–4.00). In Chen et al. (26), a longer duration of proteasome-inhibitor therapy was associated with higher odds of CV events (adjusted OR 1.94, 95% CI 1.21–3.09). Separately, Cornell et al. (25) reported a higher hazard of CV events among patients receiving carfilzomib-based therapy (HR 3.00, 95% CI 1.10–8.40). Because these studies used different effect measures, the magnitudes of these associations should not be directly compared.

#### 3.3.5 Laboratory and biomarker-related predictors

Evidence regarding biomarker-related predictors was limited to a single study (25), which reported that elevated baseline natriuretic peptide levels were associated with subsequent cardiac events (HR 4.10, 95% CI: 2.10–8.10), while a marked elevation in natriuretic peptide levels during the first cycle of therapy was associated with an even stronger increase in CV risk (HR 9.50, 95% CI: 4.30–20.70). Given the lack of external validation from additional independent cohorts, these biomarker findings were summarized narratively and require further confirmation in future prospective studies.

## 4 Discussion

In this systematic review, 20 candidate predictors of CV events in MM were identified from multivariable analyses, although only six were reported in more than one study and only one could be pooled quantitatively. Taken together, the available evidence suggests that CV risk in MM is not driven by a single factor, but rather emerges from the interaction of baseline CV vulnerability, treatment-related toxicity, and evolving markers of cardiac stress during therapy. This conceptual framework is consistent with prior reviews in cardio-oncology and MM (5, 10). At the same time, the current literature remains fragmented and methodologically heterogeneous, which limits both the strength of inference and the immediate clinical applicability of these findings.

Among the identified predictors, the most consistent signals were traditional CV risk factors, including hypertension, older age, male sex, smoking history, and pre-existing CV disease. These variables define a baseline vulnerability profile that appears highly relevant in MM. This observation is clinically important because it indicates that CV events in MM should not be viewed solely as treatment complications. Rather, many patients enter therapy with an already elevated CV risk burden, which may subsequently be amplified by disease-related and treatment-related factors. Although traditional risk factors are well established in general cardiology, their interaction with MM-specific biology remains incompletely understood. Chronic inflammation, endothelial dysfunction, renal impairment, and other systemic consequences of MM may plausibly intensify underlying CV susceptibility (2, 5), although this mechanism has not been adequately examined in prognostic studies of MM populations.

Treatment-related factors represent the second major domain of risk. In particular, carfilzomib exposure and longer duration of proteasome inhibitor therapy were associated with a higher likelihood of CV events in one retrospective study. However, this finding should be interpreted cautiously because longer treatment duration also entails a longer period at risk, and the study could not distinguish a cumulative treatment effect from increased observation time. These findings are consistent with prior evidence showing that proteasome inhibitor–associated CV toxicity may manifest as hypertension, heart failure, ischemic events, or other CV complications (5–7, 10). Importantly, the available data suggest that this risk may evolve over time rather than being limited to an immediate idiosyncratic reaction (7). This has practical implications, because it argues against a purely baseline approach to risk evaluation and supports the need for ongoing CV surveillance during treatment. However, the evidence in this area remains limited. Only one included study identified treatment duration as an independent correlate of CV events, and no included study established a clear cumulative dose–response relationship. Therefore, although treatment exposure appears to be a potentially modifiable determinant of risk, the extent to which this knowledge can currently inform management decisions remains uncertain.

Another theme emerging from this review is the possible added value of dynamic biomarkers. Limited evidence suggests that elevations in natriuretic peptides during treatment may carry greater prognostic relevance than baseline values alone, implying that serial measurements may better capture evolving myocardial stress than a single pre-treatment assessment. This interpretation is aligned with broader principles in cardio-oncology, where cardiotoxicity is increasingly understood as a progressive process that may first manifest through biomarker changes or subtle imaging abnormalities before overt clinical events occur (10, 28, 29). Serial assessment could therefore be particularly valuable in MM patients exposed to potentially cardiotoxic regimens. Nevertheless, this part of the evidence base remains underdeveloped. The number of studies is small, sample sizes are limited, and neither optimal monitoring intervals nor clinically actionable thresholds have been established. Accordingly, dynamic biomarker assessment should currently be viewed as a promising direction for risk refinement rather than a validated strategy for routine implementation in MM care.

An important implication of these findings is that the current literature largely remains at the stage of prognostic factor identification rather than individualized risk prediction. Reliable prediction models require adequate sample size, standardized endpoints, careful handling of missing data, and transparent model development and validation procedures, as emphasized by prognostic research guidance and TRIPOD-related methodological standards (30–32).

This gap is closely related to the methodological limitations of the current evidence base. First, there is considerable heterogeneity in CV outcome definitions across studies. Some studies focused on CV adverse events, others on CV mortality, and others on hypertension or composite endpoints. Such inconsistency reduces comparability across studies and limits the interpretability of pooled findings. It also poses a major obstacle to the development of prediction models, which depend on clearly defined and clinically meaningful endpoints (30). Moreover, most included studies used retrospective designs, making them vulnerable to information bias, selection bias, and incomplete ascertainment of exposures and outcomes. In administrative database studies or retrospective chart reviews, details such as actual delivered dose, treatment adherence, outpatient CV events, and subclinical cardiac abnormalities are often insufficiently captured. As a result, the reported associations may be affected by misclassification and residual uncertainty.

Several studies provided limited information on the temporal relationship between predictor assessment and outcome occurrence. This issue is particularly relevant for biomarkers and clinical parameters measured shortly before a CV event, since such variables may reflect early manifestations of evolving disease rather than true predictors useful for advance risk stratification. Confounding adjustment also varied considerably across studies. Factors such as disease stage, treatment intensity, cumulative exposure, renal function, and baseline comorbidity burden may influence both treatment selection and CV outcomes. In the absence of consistent adjustment for these variables, some reported associations may reflect residual confounding rather than independent prognostic effects. In addition, reporting on missing data was often insufficient, making it difficult to judge the extent to which incomplete information may have biased effect estimates. Finally, the predominance of small, single-center studies limits external generalizability. Reliable prognostic research and prediction model development require sufficient event numbers, standardized outcomes, and rigorous modeling and reporting practices, as emphasized by TRIPOD and related methodological guidance (30, 31). These conditions are not yet adequately met in the current MM literature.

Taken together, these considerations suggest that the present evidence is best interpreted as identifying candidate risk signals rather than establishing a clinically mature framework for CV risk stratification in MM. Even so, several practical implications can be drawn. Baseline CV assessment should remain an integral part of MM management, particularly in patients with hypertension, prior CV disease, older age, or other conventional risk factors (11, 25). Greater vigilance may also be warranted in patients receiving carfilzomib or prolonged proteasome inhibitor therapy (25). However, current data do not yet support precise thresholds for intensified monitoring, treatment modification, or prophylactic cardioprotective therapy in this population. Thus, the main clinical value of the existing evidence lies in increasing awareness of CV vulnerability and supporting closer multidisciplinary evaluation in patients judged to be at higher risk (11).

In this context, it is important to acknowledge existing risk stratification tools developed for MM. Validated scores such as IMPEDE-VTE (33) and SAVED (34) are well established for predicting VTE but are not designed to capture the broader spectrum of CVAEs examined in this review, including heart failure, arrhythmia, hypertension, and ischemic events. Beyond thrombosis-focused tools, broader cardio-oncology frameworks [e.g., HFA-ICOS (35)] have been proposed for patients receiving potentially cardiotoxic therapies; however, these frameworks have not been prospectively validated in MM-specific populations and may not fully reflect the distinctive cardiovascular risk profile associated with antimyeloma regimens. In addition, because several included studies incorporated VTE within composite CVAE endpoints, this likely contributes to outcome heterogeneity and requires cautious interpretation.

Future research should move beyond isolated association analyses toward clinically applicable risk stratification strategies. Prospective, multicenter studies with standardized CV endpoints are needed to validate existing signals and to determine how baseline factors, treatment exposure, biomarkers, imaging findings, and electrocardiographic parameters can be integrated into prediction models tailored to MM. Such models should subsequently undergo external validation and assessment of clinical utility before implementation (30, 31). In parallel, studies are needed to clarify whether biomarker-guided surveillance, imaging-based monitoring, or treatment adaptation can reduce CV events without compromising myeloma control. Addressing these gaps will be essential for transitioning from descriptive epidemiologic associations to precision CV care in MM.

## 5 Conclusion

In conclusion, cardiovascular risk in MM appears to reflect the combined influence of baseline vulnerability, treatment-related exposure, and dynamic indicators of cardiac stress. Although several candidate prognostic factors have been identified, the evidence remains limited by heterogeneous outcome definitions, predominantly retrospective designs, and inconsistent confounding adjustment. Current findings should therefore be regarded as hypothesis-generating rather than sufficient for individualized risk prediction. Prospective multicenter studies using standardized cardiovascular endpoints are needed to validate these signals and develop MM-specific cardiovascular risk prediction models.

## 6 Limitations

Several limitations should be acknowledged. The available evidence was limited by the small number of eligible studies, the predominance of retrospective designs, and marked heterogeneity in study populations, treatment settings, predictor definitions, CV endpoints, and effect measures. Consequently, only one predictor could be quantitatively pooled, whereas most findings were synthesized narratively. In addition, confounding adjustment and reporting of missing data were inconsistent across studies, and some important variables such as disease severity, renal function, and cumulative treatment exposure were not uniformly considered. Publication bias could not be formally assessed because of the small number of studies per meta-analysis. In addition, the generalizability of these findings to patients with concomitant cardiac AL amyloidosis remains uncertain, as this condition represents a distinct high-risk phenotype associated with substantially poorer survival in MM (14). Taken together, these limitations indicate that the findings of this review should be regarded as identifying candidate prognostic signals rather than providing definitive evidence for individualized CV risk prediction in multiple myeloma.

## Data Availability

The original contributions presented in the study are included in the article/Supplementary Material, further inquiries can be directed to the corresponding author.
