# Supplementary material for: Predictors of cardiovascular events in multiple myeloma: a systematic review and exploratory meta-analysis
Source: Front Cardiovasc Med. 2026 Oct 20;13:1925889. doi: 10.3389/fcvm.2026.1925889 (PMC13750087; doi:10.3389/fcvm.2026.1925889)
Supplement: Supplementary file 1 [file Table1.docx]

| **Database** | **Search Strategy** | **Final Results** |
| --- | --- | --- |
| **Cochrane Library** | #1 MeSH descriptor: [Multiple Myeloma] explode all trees  #2 ('kahler disease' OR 'morbus kahler' OR 'multiple myelomas' OR 'myeloma multiple*' OR 'myelomatoses' OR 'myelomatosis')  #3 MeSH descriptor: [Cardiovascular Diseases] explode all trees  #4 ('angiocardiovascular disease' OR 'cardiac event*' OR 'cardiovascular complication' OR 'cardiovascular disease*' OR 'cardiovascular disorder' OR 'cardiovascular disturbance' OR 'cardiovascular lesion' OR 'cardiovascular syndrome' OR 'major adverse cardiac events')  #5 Risk Factors  #6 ('population at risk' OR 'relative risk' OR 'risk score*' OR 'social risk factor*')  #7 #1 OR #2  #8 #3 OR #4  #9 #5 OR #6  #10 #7 AND #8 AND #9 | 291 |
| **EMBASE** | #1 'multiple myeloma'/exp  #2 'plasma cell myelomas':ti,ab,kw OR 'kahler disease':ti,ab,kw OR 'morbus kahler':ti,ab,kw OR 'multiple myeloma*':ti,ab,kw OR 'myeloma multiples':ti,ab,kw OR 'myeloma multiplex':ti,ab,kw OR 'myelomatoses':ti,ab,kw OR 'myelomatosis':ti,ab,kw OR 'plasma cell myeloma':ti,ab,kw  #3 #1 OR #2  #4 'cardiovascular disease'/exp  #5 'cardiovascular diseases':ti,ab,kw OR 'adverse cardiac event*':ti,ab,kw OR 'angiocardiopathy':ti,ab,kw OR 'angiocardiovascular disease':ti,ab,kw OR 'cardiac event*':ti,ab,kw OR 'cardiovascular complication':ti,ab,kw OR 'cardiovascular disease*':ti,ab,kw OR 'cardiovascular disorder':ti,ab,kw OR 'cardiovascular disturbance':ti,ab,kw OR 'cardiovascular lesion':ti,ab,kw OR 'cardiovascular syndrome':ti,ab,kw OR 'major adverse cardiac events':ti,ab,kw  #6 #4 OR #5  #7 'risk factor'/exp  #8 'health correlates':ti,ab,kw OR 'population at risk':ti,ab,kw OR 'relative risk':ti,ab,kw OR 'risk factor*':ti,ab,kw OR 'risk score*':ti,ab,kw OR 'social risk factor*':ti,ab,kw  #9 #7 OR #8  #10 #3 AND #6 AND #9 | 1995 |
|  |  |  |
| **Ovid Medline** | #1 Multiple Myeloma/  #2 (Plasma Cell Myelomas or kahler disease or morbus kahler or multiple myelomas or myeloma multiple* or myelomatoses or myelomatosis).ab,ti,kw.  #3 Cardiovascular Diseases/  #4 (adverse cardiac event* or angiocardiopathy or angiocardiovascular disease or cardiac event* or cardiovascular complication or cardiovascular disease* or cardiovascular disorder or cardiovascular disturbance or cardiovascular lesion or cardiovascular syndrome or major adverse cardiac events).ab,ti,kw.  #5 Risk Factors/  #6 (health correlates or population at risk or relative risk or risk score* or social risk factor*).ab,ti,kw.  #7 #1 OR #2  #8 #3 OR #4  #9 #5 OR #6  #10 #7 AND #8 AND #9 | 30 |
| **PubMed** | (((Multiple Myeloma[MeSH Terms]) OR (("kahler disease"[Title/Abstract] OR "Plasma Cell Myelomas"[Title/Abstract] OR "kahler disease"[Title/Abstract] OR "morbus kahler"[Title/Abstract] OR "multiple myeloma*"[Title/Abstract] OR "myeloma multiplex"[Title/Abstract] OR "myelomatoses"[Title/Abstract] OR "myelomatosis"[Title/Abstract] OR "plasma cell myeloma"[Title/Abstract]))) AND (("CARDIOVASCULAR DISEASES"[Title/Abstract] OR "Adverse Cardiac Event*"[Title/Abstract] OR "angiocardiopathy"[Title/Abstract] OR "cardiac event*"[Title/Abstract] OR "cardiovascular complication"[Title/Abstract] OR "cardiovascular disease*"[Title/Abstract] OR "cardiovascular disorder"[Title/Abstract] OR "cardiovascular disturbance"[Title/Abstract] OR "cardiovascular lesion"[Title/Abstract] OR "cardiovascular syndrome"[Title/Abstract] OR "major adverse cardiac events"[Title/Abstract]) OR (Cardiovascular Diseases[MeSH Terms]))) AND ((("health correlates"[Title/Abstract] OR "population at risk"[Title/Abstract] OR "relative risk"[Title/Abstract] OR "risk factor*"[Title/Abstract] OR "risk score*"[Title/Abstract] OR "Social Risk Factor*"[Title/Abstract]) OR (influencing factor[Title/Abstract])) OR (Risk Factors[MeSH Terms])) | 2336 |
|  |  |  |
| **Web of Science** | #1 Multiple Myeloma (All Fields)  #2 ("kahler disease" OR "morbus Kahler" OR "multiple myelomas" OR "myeloma multiple*" OR "myelomatoses" OR "myelomatosis") (All Fields)  #3 Cardiovascular Diseases (All Fields)  #4 ("adverse cardiac event*" OR "angiocardiopathy" OR "angiocardiovascular disease" OR "cardiac event*" OR "cardiovascular complication" OR "cardiovascular disease*" OR "cardiovascular disorder" OR "cardiovascular disturbance" OR "cardiovascular lesion" OR "cardiovascular syndrome" OR "major adverse cardiac events") (All Fields)  #5 Risk Factors (All Fields)  #6 ("health correlates" OR "population at risk" OR "relative risk" OR "risk score*" OR "social risk factor*") (All Fields)  #7 #1 OR #2  #8 #3 OR #4  #9 #5 OR #6  #10 #7 AND #8 AND #9 | 134 |
