# Supplementary material for: Predictors of cardiovascular events in multiple myeloma: a systematic review and exploratory meta-analysis
Source: Front Cardiovasc Med. 2026 Oct 20;13:1925889. doi: 10.3389/fcvm.2026.1925889 (PMC13750087; doi:10.3389/fcvm.2026.1925889)
Supplement: Supplementary file 2 [file Table2.docx]

| Domain | Inclusion Criteria | Exclusion Criteria |
| --- | --- | --- |
| Study Population | -Adult patients (≥ 18 years old).  -Diagnosed according to the 2014 IMWG criteria, contemporaneous diagnostic criteria, or validated registry/administrative definitions used in the original study. | -Patients with concurrent cardiac amyloidosis (whether clinically highly suspected or histologically confirmed).  - Animal models or in vitro studies. |
| Predictors / Exposure | -Studies explicitly investigating  ≥ 1 well-defined predictor (demographic, clinical, treatment, or laboratory) for cardiovascular (CV) events. | -Studies not focused on identifying or evaluating predictors for CV events |
| Outcomes | -Clearly defined CV events (e.g., myocardial infarction, heart failure, arrhythmias).  -Provided quantitative risk estimates (e.g., Hazard Ratio [HR], Odds Ratio [OR]) derived from multivariable analysis. | -Insufficient outcome data to evaluate risk.  -Studies reporting only univariable analysis without multivariable adjustments for CV predictors |
| Study Design / Article Type | -Primary clinical studies (including prospective or retrospective cohorts, case-control studies, and randomized controlled trials [RCTs]). | Non-primary literature (reviews, scoping/systematic reviews, case reports).  -Editorials, conference abstracts, posters, and opinion pieces.  -Duplicate publications |
| Language | Full-text articles available in English | Full-text articles not available in English |
