# Supplementary material for: Predictors of cardiovascular events in multiple myeloma: a systematic review and exploratory meta-analysis
Source: Front Cardiovasc Med. 2026 Oct 20;13:1925889. doi: 10.3389/fcvm.2026.1925889 (PMC13750087; doi:10.3389/fcvm.2026.1925889)
Supplement: Supplementary file 3 [file Table3.docx]

| No. of studies | Study design | Risk of bias | Inconsistency | Indirectness | Imprecision | Publication bias | Certainty | Pooled effect (95%CI) |
| --- | --- | --- | --- | --- | --- | --- | --- | --- |
| 2 | Observational cohort studies ^(a)^ | Serious ^(a)^ | Not serious ^(b)^ | Serious ^(c)^ | Not serious ^(d)^ | Cannot be assessed ^(e)^ | Low | HR 1.90 (1.57‑2.29) |

Table S3. GRADE Summary-of-Findings for the exploratory meta-analysis of hypertension as a predictor of composite cardiovascular adverse events

Note. Adapted GRADE framework for prognostic evidence; judgements based on QUIPS risk‑of‑bias tool for prognostic studies. Abbreviations: CVAEs, cardiovascular adverse events; CI, confidence interval; HR, hazard ratio; QUIPS, Quality in Prognosis Studies.

^(a)^ Rated down for risk of bias: included observational studies had moderate concerns regarding confounding adjustment according to QUIPS tool.

^(b)^ Not rated down for inconsistency: I² = 0%, P for heterogeneity = 0.83; consistent direction of effect across studies.

^(c)^ Rated down for indirectness: composite CVAEs definitions varied across primary studies, with variable inclusion of venous thromboembolism and diverse cardiovascular manifestations; additionally, hypertension definitions differed (baseline pre‑existing versus treatment‑emergent hypertension).

^(d)^ Not rated down for imprecision: 95% confidence interval did not cross the null value of 1.0, and the interval was relatively narrow.

^(e)^ Publication bias could not be reliably assessed, given fewer than 10 included studies.
